# An assessment of the laboratory network in Ghana: A national-level ATLAS survey (2019–2020)

**DOI:** 10.4102/ajlm.v12i1.1844

**Published:** 2023-02-08

**Authors:** Emma E. Kploanyi, Joseph Kenu, Benedicta K. Atsu, David A. Opare, Franklin Asiedu-Bekoe, Lee F. Schroeder, David W. Dowdy, Alfred E. Yawson, Ernest Kenu

**Affiliations:** 1 School of Public Health, University of Ghana, Legon, Accra, Ghana; 2 National Public Health and Reference Laboratory, Ghana Health Service, Accra, Ghana; 3 Public Health Division, Ghana Health Service, Accra, Ghana; 4 Department of Pathology and Clinical Laboratories, University of Michigan, Ann Arbor, Michigan, United States; 5 Department of Epidemiology, Johns Hopkins Bloomberg School of Public Health, Baltimore, Maryland, United States; 6 Department of Community Health, University of Ghana Medical School, Accra, Ghana

**Keywords:** laboratory systems, laboratory network, ATLAS, epidemic-prone diseases, LABNET scorecard, laboratory capacity, laboratory readiness, laboratory strengthening

## Abstract

**Background:**

Integrated health systems with strong laboratory networks are critical in improving public health. The current study assessed the laboratory network in Ghana and its functionality using the Assessment Tool for Laboratory Services (ATLAS).

**Intervention:**

A national-level laboratory network survey was conducted among stakeholders of the Ghanaian laboratory network in Accra. Face-to-face interviews were conducted from December 2019 to January 2020, with follow-up phone interviews between June and July 2020. Also, we reviewed supporting documents provided by stakeholders for supplementary information and transcribed these to identify themes. Where possible, we completed the Laboratory Network scorecard using data obtained from the ATLAS.

**Lessons learnt:**

The Laboratory Network (LABNET) scorecard assessment was a valuable addition to the ATLAS survey as it quantified the functionality of the laboratory network and its overall advancement toward achieving International Health Regulations (2005) and Global Health Security Agenda targets. Two significant challenges indicated by respondents were laboratory financing and delayed implementation of the Ghana National Health Laboratory Policy.

**Recommendations:**

Stakeholders recommended a review of the country’s funding landscape, such as funding laboratory services from the country’s internally generated funds. Also, they recommended laboratory policy implementation to ensure adequate laboratory workforce and standards.

## Background

Integrated health systems with effective and efficient laboratory networks^1,2^ are gaining wide recognition for their critical role in managing high-burden endemic infections, particularly HIV, tuberculosis and malaria,^3,4^ and accelerating disease outbreak response. The importance of laboratory outbreak response has been highlighted by the severe acute respiratory syndrome (SARS, 2003), H1N1 (2009), meningitis (2009), cholera (2010), Middle East respiratory syndrome (2012), Ebola virus disease (2014), Zika virus disease (2015), and yellow fever (2016–2017) outbreaks, as well as the current coronavirus disease 2019 (COVID-19) pandemic.^5,6,7,8,9^ There are considerable delays in outbreak detection and communication for most infectious disease outbreaks originating from Africa,^10,11^ as exemplified in the delayed response to the West African Ebola outbreak, which was reported four months after the index infection and, more recently, the delayed diagnosis of suspected COVID-19 cases in Africa.^6,12,13^ Also, the COVID-19 pandemic has underscored the diagnostic capacity challenges facing African countries. As of January 2022, Ghanaian SARS coronavirus 2 (SARS-CoV-2) daily testing rates was around 70 per 1000 people, in contrast to over 2300 per 1000 people in the United States.^14^

Improving laboratory networks is a critical step toward controlling health emergencies.^15^ The Global Health Security Agenda (GHSA) requires countries to establish tiered national laboratory systems and ‘determine an appropriate level of diagnostic capability at each level of the public health hierarchy from national to the district’.^16^

The laboratory network in Ghana was assessed in 2006, focusing on HIV diagnostic resources, using the Assessment Tool for Laboratory Services (ATLAS).^17^ The assessment identified the need for guidelines in biosafety, inventory control and logistics management information systems, and the application of policies and procedures.^18^ However, there have been no publicly available assessments of the laboratory network in Ghana since then. In this study, we conducted the ATLAS survey with a wider scope, including a Laboratory Network (LABNET) scorecard evaluation.^19^

The ATLAS survey was conducted using the central-level questionnaire to describe the national laboratory network in terms of its organisational structure, management of laboratory services and logistics, as well as quality regulation. A portion of the LABNET evaluation was added to provide a quantitative measure of functionality to corroborate the qualitative findings from the ATLAS survey on core capabilities. The assessment was conducted to identify strengths and weaknesses of the laboratory network and provide recommendations for its improvement.

## Description of the intervention

### Ethical considerations

The study obtained ethical approval from the Ghana Health Service Ethical Review Committee (GHS-ERC 005/05/19) and Noguchi Memorial Institute for Medical Research Institutional Review Board (FWA 00001824). In addition, the Director-General of the Ghana Health Service (GHS) granted permission before the study was conducted. Written consent was obtained from every participant before conducting an interview.

### Study design and setting

This was a qualitative study conducted among key stakeholders in the laboratory network in Accra, Ghana. It is part of a more extensive, ongoing study to map and model the laboratory network in Ghana for three epidemic-prone diseases (EPDs) – bacterial meningitis, measles and yellow fever – and three diseases of public health importance (DPHI): HIV, tuberculosis and hepatitis C virus (HCV). These diseases guided our choice of stakeholder interviewed.

### Data collection

We had a consultation with stakeholders from the National Public Health & Reference Laboratory (NPHRL) and Disease Surveillance Department of GHS in November 2019 to adapt the national-level United States Agency for International Development ATLAS^20^ to suit the Ghanaian context, as recommended in the ATLAS guidelines. We identified stakeholders to be interviewed with the ATLAS during the consultation session. The ATLAS comprises nine sections: organisation, policy, forecasting and procurement, financing, storage and distribution, inventory control system, laboratory information management system, supervision, and general questions.

Face-to-face interviews were conducted from December 2019 to January 2020, with follow-up phone interviews conducted between June 2020 and July 2020. Also, supporting documents provided by stakeholders were reviewed for supplementary information, and responses were transcribed to identify themes.

Where possible, data were used to complete the LABNET scorecard.^19^ The LABNET scorecard has been validated for assessing a country’s laboratory network functionality in implementing the International Health Regulations (2005) and attaining the GHSA targets.^19^ Our LABNET scorecard assessment was based on five of the nine core capabilities closely related to aspects of the laboratory network assessed by the ATLAS: political, legal, regulatory and financial framework; structure and organisation of the laboratory networks; laboratory information (management system); infrastructure, equipment and supplies; and quality of laboratory services. The scores were converted to percentages reflecting overall advancement toward standards or targets, and interpretations were drawn on each component’s functionality stage based on the weakest score (Supplementary Table 1).

### Survey implementation

A total of 12 respondents were interviewed: two representatives from Clinical Laboratories Unit (CLU) under the Institutional Care Division, GHS; Deputy Directors at Supplies, Stores and Drugs Management (SSDM) and the Finance Division, GHS; the Head and Quality Manager for NPHRL; and a representative each from the Health Facilities Regulatory Agency and Allied Health Professionals Council. Relevant stakeholders from disease control programmes including the Expanded Programme of Immunisation (EPI) with focus on EPDs, National Tuberculosis Control Programme (NTP), National AIDS and Sexually Transmitted Infections Control Programme (NACP) and National Viral Hepatitis Control Programme (NVHCP) were also interviewed.

## Results

### Organisational structure of clinical and public health laboratories

**FIGURE 1:**
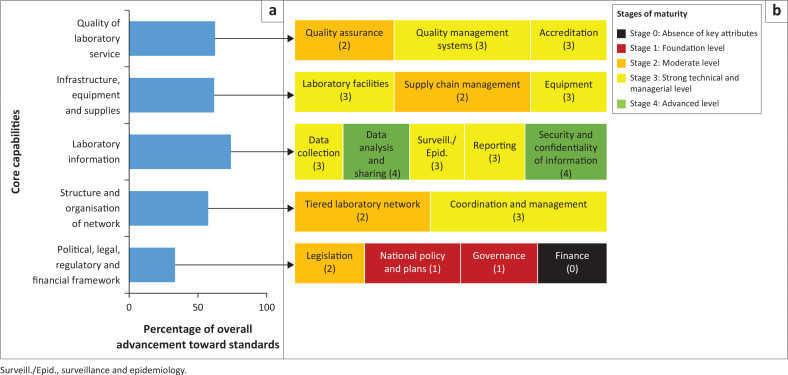
Organisational structure of clinical and public health laboratories in Ghana as of January 2020. The ATLAS assessment provided the number of laboratory facilities in each category in January, 2020.

The GHS tiers into the national, regional, district and sub-district levels with the CLU coordinating vertical laboratory activities (Figure 1). Respondents reported over 800 public clinical laboratories, including 10 regional, 112 district and about 692 sub-district health facility laboratories. Based on data from the national database District Health Information Management System II (DHIMS2) (Ghana Health Service with technical assistance from the University of Oslo Health Informatics Department, Accra, Ghana), the GHS laboratories conduct biochemistry (*n* = 342), haematology (*n* = 397) and microbiology tests (*n* = 365). The Public Health Division of the GHS is responsible for the Public Health Laboratories (PHLs) that surveil measles, rubella, yellow fever, tuberculosis, HIV and other diseases. The NPHRL in Accra has oversight responsibility for three other PHLs situated in Tamale (Northern zone), Kumasi (Middle zone), and Sekondi (Western zone).

Laboratories in the GHS organisational structure are tiered into three levels: tertiary (NPHRL and teaching hospital laboratories), secondary (regional hospital laboratories and zonal PHLs) and primary laboratories (laboratories in district, sub-district facilities and health centres). The relationship between tiers is based on referrals, flowing from lower-tier to higher-tier laboratories, with higher-tier facilities assigned some oversight responsibilities on lower-tier laboratories (e.g., outreach training and supportive supervision [OTSS]). The four teaching hospitals, although directly under Ministry of Health (MoH) but not GHS, only play a key role in the referral system. The regional laboratory scientists also serve as laboratory coordinators in various regions. However, the districts lack such coordinators due to inadequate staff capacity.

The EPI, NACP, NTP and NVHCP collaborate with clinical laboratories and PHLs to conduct laboratory testing. The EPI has outsourced all laboratory services and logistics management for EPDs to NPHRL. While the NACP has ART sites at 488 facilities, including laboratories, the NTP works with about 337 laboratories in the health system. The NPHRL coordinates national-level activities of NVHCP, whereas the health directorates coordinate activities at the regional and district levels. There are no specific laboratories designated for HCV testing under the current programme.

### Policies and other guidelines

**FIGURE 2:**
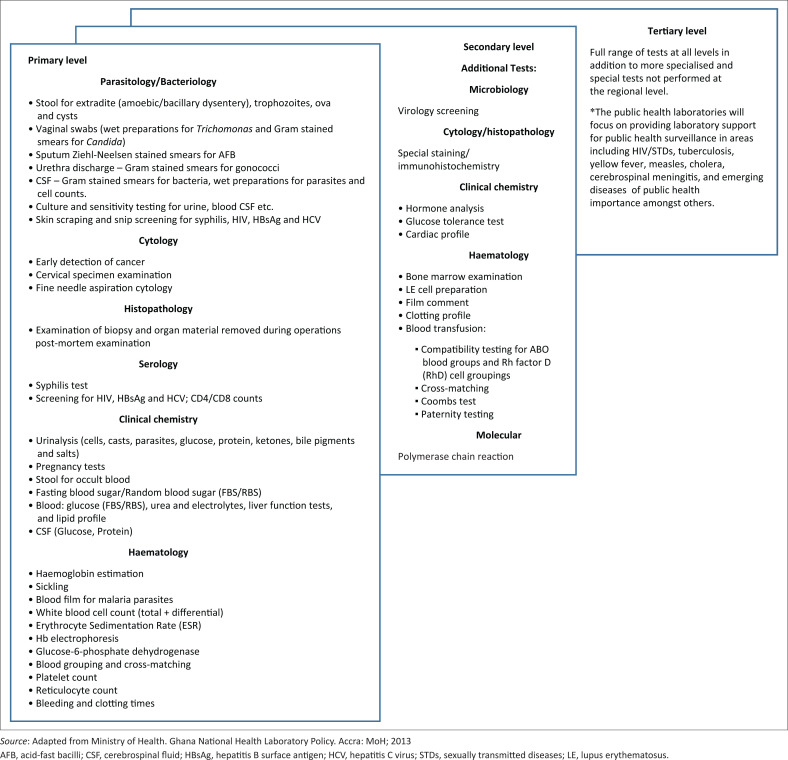
Test menu for primary, secondary and tertiary tiers in the Ghanaian laboratory network, 2020.

The MoH had no dedicated laboratory policy development unit at the time of assessment. Thus, the CLU represents the GHS on clinical laboratory policy issues. The Laboratory Technical Committee comprising members from all agencies of MoH and GHS developed the Ghana National Health Laboratory Policy^21^ that was approved in 2013; however, the Ghana National Health Laboratory Policy is still not operational. The policy describes the laboratory organisation, services and test menu by level, staffing norms, logistics management, quality management and laboratory information system. The primary level test menu covers basic parasitology and bacteriology, cytology, histopathology, serology, clinical chemistry and haematology tests. In contrast, the secondary level test menu covers more sophisticated testing such as immunohistochemistry and nucleic acid testing. Finally, the tertiary level test menu covers more specialised tests and the PHLs focus on EPD-DPHIs in support of public health surveillance (Figure 2).

The policy document on infection prevention and control^22^ is fully operational. It contains guidelines that cover infection prevention and biosafety. The PHLs perform tests using standard operating procedures (SOPs). Also, the clinical laboratories conform to international and funding programme standards. However, harmonised general clinical testing is lacking, particularly the use of different automated equipment at the facility level for haematology, immunology and chemistry.

### Financing

Funding for laboratory services in the country is fragmented for infrastructure, supplies and equipment. There are different funding sources for target programmes and PHLs; general clinical testing relies on internally generated funds and National Health Insurance System (NHIS) reimbursement on diagnostic services and tests listed on the NHIS benefits package.^23^

**FIGURE 3:**
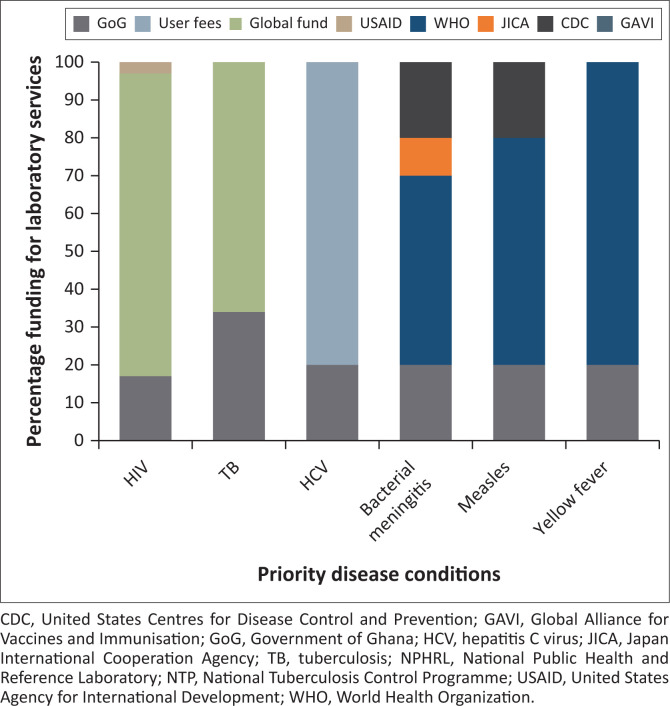
Financing of laboratory services for priority disease conditions in Ghana, 2020.

The government of Ghana only provides funding for infrastructure, some equipment and workers’ salaries. The primary funding donors were the Global Fund (Figure 3), which provided the most funding for HIV and tuberculosis, and the World Health Organization (WHO), which funds EPDs. Currently, no donor funding is available for HCV laboratory services, NHIS covers only HCV screening and remaining costs are borne by patients. Stakeholders estimated a 20% – 50% gap in funding for the six EPD-DPHIs.

Different units and divisions coordinate programme-specific testing for EPD-DPHIs. Most programmes procure laboratory supplies at the central level rather than allocate funds to laboratories. The NPHRL receives most supplies and funds from donors, allocating these to the zonal PHLs through their divisional heads. Outbreaks also determine the allocation of financial resources. Target programmes and the NPHRL have separate budgetary line items for laboratory services, supplies and equipment. However, the finance unit of the GHS works with a highly aggregated budget, the Medium Term Expenditure Framework plan for MoH^24^; hence, there is no specific budget line for laboratory services. Since Ghana attained lower-middle-income status, a transition plan was implemented in 2015, requiring the government to increase funding each year with a full transition scheduled in 2022.

### Forecasting and procurement

The target programmes, NPHRL and health facilities consistently forecast and procure needed laboratory supplies; however, there is no standardised forecasting method. The EPI does not prepare forecasts for EPDs because they have outsourced this role to NPHRL. The situation is quite peculiar for NVHCP as they also prepare forecasts but do not receive funding for laboratory supplies.

Unless otherwise requested by funders, the *Public Procurement Act 663* guides procurement as amended in 2016. For example, at NTP, a procurement request to SSDM initiates an open tender with the selection of vendors by the Central Tender Committee. A diverse stakeholder team monitors the procurement process. The average lead time is programme-specific, being six months for NACP and inconsistent for NTP due to time lags between contract award and item supply. Although the programmes operate independently, there are instances when they carry out joint activities, especially between the NTP and NACP. Their procurement units lead procurement and monitoring for general clinical testing of their target disease conditions at the facilities.

All programmes indicated adequate laboratory supplies, except the NVHCP, which has no funding for laboratories. The NACP supplies reagents and haematology and chemistry analysers to the ART sites, whereas the NTP supplies reagents to all tuberculosis testing laboratories. However, there was little information on the adequacy of supplies at the PHLs and clinical laboratories, as this assessment was conducted only at the national level. Although multiple systems manage laboratory supplies, respondents reported that duplicating efforts had been minimised.

### Storage and distribution

The Integrated Scheduled Delivery System implements the Last Mile Distribution Strategy of laboratory supplies and equipment to all levels other than PHLs. Currently, there are four storage facilities for laboratory supplies and cold chain reagents at the central level, and although these storage facilities have adequate cold chain capacity, the regional stores do not. Thus, a central medical store is being built for future storage needs. A third-party logistics firm is contracted for distribution as GHS delivery vehicles are insufficient. The NPHRL handles supplies at the national level for onward distribution to the zonal PHLs. The programmes intervene when there is a need for redistribution or emergency distribution.

### Inventory control

The International Organization for Standardization (ISO) 15189 requires establishing an inventory system with preset minimum and maximum stock levels. Hence, SOPs have been developed by the SSDM indicating the minimum, reorder points and maximum stock levels for facilities. The minimum and maximum stock levels are two and three months for the central level and three and six months for the regional medical stores. The laboratory facilities determine order quantities for general clinical testing supplies, higher-level authorities at the central level for programmes, and the head of NPHRL for all PHLs. The laboratory scientists at the facilities collaborate with their procurement units to conduct stock-taking twice annually, using various mechanisms, such as WhatsApp platforms (Meta Platforms, Menlo Park, California, United States), Microsoft Excel spreadsheets (Microsoft, Redmond, Washington, United States), and Gx-Alerts (Cepheid, Sunnyvale, California, United States), aside from the monthly stocks reports. The WHO monitors stock for measles, rubella and yellow fever through kit management reports regularly submitted by the NPHRL, while the NPHRL monitors activities, including stock balances, at the zonal PHLs.

### Specimen transport system

Based on the current testing algorithm, sputum samples from all new suspected cases are tested using GeneXpert^®^ machines (Cepheid, Sunnyvale, California, United States). Thus, the available 127 GeneXperts^®^ are strategically placed within a 2-h drive to most facilities, easing specimen referral. The Ghana Postal Courier Service is contracted to transport samples on specific pickup days, ensuring collection within two days. Tuberculosis drug resistance testing is performed at five laboratories, with specimen referral arranged by the requesting facility through public transport or courier service. HIV viral load testing and early infant diagnosis are performed at 10 regional and four teaching hospitals. As with tuberculosis diagnosis, the courier service transports HIV specimens on facility-specific pickup days. The requesting facility arranges specimen transport for all other specimen referrals.

### Laboratory information system management

**FIGURE 4:**
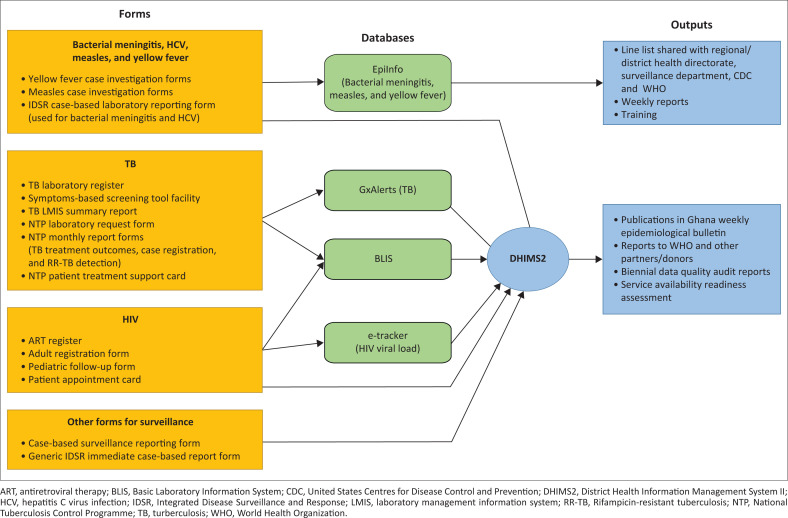
Reporting system for laboratory services management information in Ghanaas of 2020.

The health information management system used in all health facilities is the DHIMS2. This database is an upgrade from the DHIMS, which incorporates tracking by target programmes and improves cause-of-death statistics. Like most clinical databases, the DHIMS2 captures laboratory service data at an aggregate level down to facility, but not patient level. The basic laboratory information system also reports into DHIMS2 periodically. Similarly, e-tracker data for HIV viral load are captured in the DHIMS2. Two other databases do not directly report data into DHIMS2: Gx-Alert for GeneXpert, and EpiInfo (Epi Info™, Centers for Disease Control and Prevention, Atlanta, Georgia, United States) or data on EPDs. Gx-Alert data are aggregated at the facility level and entered manually into DHIMS2 whereas EpiInfo captures data on the WHO disease network that are reported directly to the WHO. No entries are made into DHIMS2 (Figure 4).

The PHLs prepare weekly yellow fever reports and communicate positive results via phone. Other reports are submitted either electronically or as hard copies or by both methods. Standard national forms used to collect and report data into DHIMS2 include case-based forms and other national forms developed by the CLU for haematology, chemistry and microbiology testing. They also provide demographic data crucial for planning immunisation activities. There are also logbooks at the laboratories that provide information on the laboratory tests requested and conducted. However, only aggregated data are available at the national level.

As described, reports from these databases are useful for quantifying commodities during forecasting, procurement, inventory control, targeted screening, planning public health interventions, notifying international organisations on disease burden and outbreaks, and seeking donor support. However, not all facilities submit data as and when due. The NPHRL monitors reporting rates and supports supervision exercises organised by PHLs.

### Supervision

The CLU does not supervise private laboratories. Instead, it carries out supportive supervision for general clinical testing at the regional level. Another regional-level team supervises the district and sub-district levels. In addition, the CLU performs supervisory visits every six months using the OTSS checklist for malaria diagnosis and the Integrated Monitoring and Supervision Checklist for general supervision.

For the PHLs, a national-level supervisory team monitors reagent availability, storage and shelf life, and staff competencies and credentials using checklists. Supervision is quarterly at the NPHRL but less frequent at the zonal PHLs due to insufficient logistics; however, the laboratory managers monitor daily activities.

Target programmes, mainly NACP and NTP, organise supervisory visits to laboratories every quarter or twice annually, depending on resources. These programmes are building local capacity so that representatives can supervise activities at the district level. The EPI only provides supervision and support for the national and regional cold rooms.

### Quality regulation and accreditation

At the time of assessment, no laboratory had been ISO accredited. However, the CLU has assessed some laboratories preparing for accreditation using the Stepwise Laboratory Improvement Process Towards Accreditation checklist.^25^ The CLU also carries out external quality assurance through OTSS (Supplementary Figure 1), supporting the implementation of ISO 15189. The NPHRL and zonal PHLs have implemented quality management systems based on this standard with self-regulation.

The Health Facilities Regulatory Agency under the MoH regulates laboratory quality independently of the CLU. They issue laboratory operation licenses based on an intensive checklist inspection, with tiered accreditation: 50% (6-month license) or 90% (3-year renewal) of items. Unannounced laboratory monitoring is carried out twice annually and could lead to a reassignment of the tier, influencing the laboratory’s NHIS reimbursement.

The Allied Health Professionals Council regulates laboratory professionals through licensing examinations and sanctions. Although the Council is enjoined by Act 857 to supervise practitioners, this is lacking due to fund constraints. Instead, their interventions are responses to malpractice brought to their attention.

### The assessment of laboratory network functionality using the Laboratory Network scorecard

**FIGURE 5:**
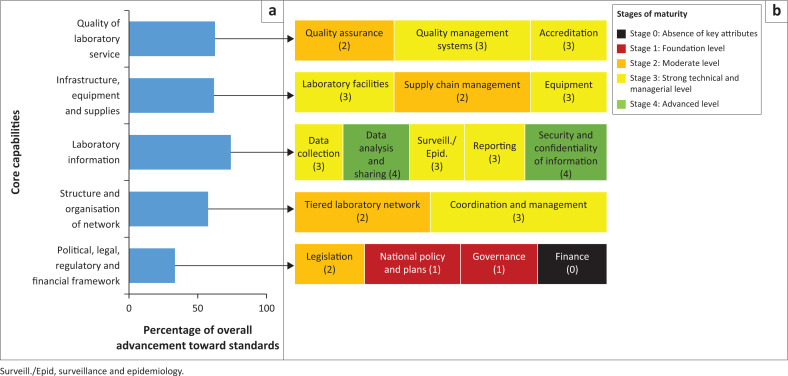
Country results for assessment of the laboratory network in Ghana using the LABNET scorecard, 2020. (a) Overall advancement of core capabilities towards the standards taking all scores into consideration; (b) stages of functionality highlighting weak scores.

The self-report of stakeholders on five of the nine core capabilities of the laboratory network using the LABNET scorecard indicated that the laboratory information management system advanced the most (74%) toward achieving the International Health Regulations (IHR) and GHSA targets (Figure 5; Supplementary Table 1). Three capabilities were similarly rated: structure and organisation of the laboratory networks (58%), infrastructure, equipment and supplies (62%), and quality of the laboratory services (63%). However, the political, legal, regulatory and financial framework rated the lowest (33%), with the finance component at the least functional stage as it lacked vital attributes.

## Lessons learnt

### Experiences

Although ATLAS guidelines recommend group discussions for administering the tools, we adopted key informant interviews due to stakeholders’ conflicting and limited availability for a group discussion. Multiple stakeholders with in-depth knowledge and experience in one or more tool sections were interviewed to obtain a holistic picture of the current laboratory network. This mode of administering the tool offered the added advantage of receiving more detailed responses than obtained in a time-limited group discussion. Moreover, the key informant interviews served as a basis to receive further information from respondents to consolidate findings. Some respondents recommended other stakeholders that could better respond to some sections of the tool; hence, the earlier list of stakeholders generated from the consultative meeting was updated a few times. The LABNET scorecard assessment was a valuable addition to the ATLAS survey as it provided quantitative responses that served as a reflection of the overall advancement toward standards and targets set by IHR (2005) and GHSA.

Based on the experiences described, we drew lessons, and identified some strengths and challenges:

### Lessons

- The ATLAS was a useful baseline assessment of the entire laboratory network because multiple stakeholders with in-depth knowledge and experience in one or more tool sections were interviewed.
- The LABNET scorecard assessment was a useful addition to the ATLAS survey as it assessed the functionality of the laboratory network through quantitative responses that served as a reflection of the overall advancement toward targets set by IHR (2005) and GHSA.
- The lowest LABNET score was obtained in the ‘Legal and regulatory framework’ assessment; the Ghana National Health Laboratory Policy although approved has not yet been implemented, driving the lack of harmonised test menu, reagents and equipment. Thus, the laboratory policy needs to be implemented to ensure an adequate workforce and standardisation of laboratories. A weak legal and regulatory framework impacts all other LABNET core components and must be addressed first.
- Vertical programmes rely heavily on external funding, whereas health facilities solely rely on internally generated funds for laboratory supply procurement and service delivery. This presents the opportunity for a better collaboration with vertical programmes to access external funding or adapt the funding mechanisms of health facilities through targeted policy and administrative interventions.
- The supervision and quality regulation organised by the administrative units and vertical programmes align with ISO standards and could be leveraged for the accreditation of laboratories.

### Strengths

The assessment found a collaboration between disease control programmes, clinical and PHLs in the forecasting, procurement and distribution of laboratory supplies, testing for priority target disease conditions, and supportive supervision for laboratory testing. The assessment also found NHIS reimbursement on diagnostic services and tests listed on the benefits package as an opportunity to increase local financing of laboratory services.

Some gaps identified by the first ATLAS assessment^18^ have been addressed over the years through the development and implementation of the Policy for Infection Prevention and Control, and SOPs guiding inventory control and laboratory testing. It is commendable that the country has a national database into which data from clinical and PHLs are reported and easily accessed for decision-making. The LABNET assessment indicated that the laboratory information management system advanced the most toward achieving IHR and GHSA targets.

The specimen transport arrangements between some target programmes (NTP and NACP) and Ghana Postal Courier Service serve as a good foundation to developing a specimen referral and transport system for all priority conditions.

### Challenges

Laboratory funding was a major challenge indicated by most respondents. Target programmes rely heavily on external funding; the CLU reported that the internally generated funds and the often-delayed NHIS reimbursements were insufficient to maintain testing capacity (Figure 3). Funding from the government of Ghana was insufficient for laboratory services, and there was no specific budget line for these services at the central level. Inadequate funds at the central level affect holistic and integrated supervision. Currently, the CLU takes advantage of the OTSS for malaria diagnosis to conduct general supervision. Three out of five stakeholders from CLU, NPHRL and EPI indicated that some laboratories run out of reagents and other supplies due to insufficient funds. The NVHCP has especially suffered from inadequate funding for its activities. Programmes that receive external funding do not have the liberty of discretionary expending as funders dictate spending.

Another challenge is the delayed implementation of the Laboratory Policy approved in 2013. As a result, test menu harmonisation and reagents and equipment standardisation are a challenge, affecting specimen and patient referral to higher tiers. In addition, there is no organised system at the national level for sample transportation except for HIV and tuberculosis diagnoses.

### Study limitations

This assessment was carried out to characterise the laboratory network in Ghana using the ATLAS, a qualitative tool. In addition, some aspects of the LABNET scorecard were used to measure functionality quantitatively in support of findings on five out of the nine core capabilities that were related to some aspects of the ATLAS administered.

Ideally, the entire qualitative and quantitative LABNET evaluation should be applied as part of a multisectoral workshop led by the MoH, with results based on consensus. Thus, future assessments should consider administering the entire LABNET tool and convene workshops to validate findings.

### Conclusion

Laboratories under the Ghana Health Service are tiered into three levels with the clinical laboratories vertically coordinated by the CLU whereas the Public Health Unit coordinates the PHLs. The laboratories collaborated with target programmes in the forecasting, procurement and distribution of laboratory supplies, supportive supervision and testing for priority conditions. No laboratory had yet received ISO accreditation at the time of assessment although some had been assessed using the Stepwise Laboratory Improvement Process Towards Accreditation checklist. In terms of functionality, the laboratory information management system advanced the most toward achieving IHR and GHSA targets whereas the political, legal, regulatory and financial framework lagged behind with the finance component at the lowest stage of functionality. Major gaps were identified in laboratory financing and the implementation of the National Health Laboratory Policy. These should represent the focus of future initiatives to strengthen the laboratory network in Ghana.

## Recommendations

Stakeholders recommended that the funding landscape for the country be reviewed. Considerations should be made to fund laboratory services from the country’s internally generated funds and expand the scope of NHIS. Also, the MoH should create a laboratory unit to collaborate with administrative units for laboratories in all their agencies to update and implement the National Health Laboratory Policy to guide improvement of the laboratory network towards achieving the GHSA targets. Moreover, plans for a national sample referral and transportation system that accommodate a wide range of clinical testing needs should be operationalised. The collaboration between administrative units and target programmes could be improved through joint supervision to optimise the resources available. Also, the quality regulation in laboratories align with ISO standards and could be leveraged for the accreditation of laboratories. A follow up LABNET assessment could be conducted to help document the laboratory network’s progress towards attaining the GHSA targets on all nine core capabilities.
